# NFkB is essential for activin-induced colorectal cancer migration via upregulation of PI3K-MDM2 pathway

**DOI:** 10.18632/oncotarget.16343

**Published:** 2017-03-18

**Authors:** Arundhati Jana, Nancy L Krett, Grace Guzman, Ahmer Khalid, Ozkan Ozden, Jonas J Staudacher, Jessica Bauer, Seung Hyun Baik, Timothy Carroll, Cemal Yazici, Barbara Jung

**Affiliations:** ^1^ Division of Gastroenterology and Hepatology, University of Illinois Medical College, Chicago, IL 60612, USA; ^2^ Department of Pathology, University of Illinois Medical College, Chicago, IL 60612, USA

**Keywords:** activin, NFkB, colon cancer, MDM2, migration

## Abstract

Colorectal cancer (CRC) remains a common and deadly cancer due to metastatic disease. Activin and TGFB (TGFβ) signaling are growth suppressive pathways that exert non-canonical pro-metastatic effects late in CRC carcinogenesis. We have recently shown that activin downregulates p21 via ubiquitination and degradation associated with enhanced cellular migration independent of SMADs. To investigate the mechanism of metastatic activin signaling, we examined activated NFkB signaling and activin ligand expression in CRC patient samples and found a strong correlation. We hypothesize that activation of the E3 ubiquitin ligase MDM2 by NFkB leads to p21 degradation in response to activin treatment. To dissect the link between activin and pro-carcinogenic NFkB signaling and downstream targets, we found that activin but not TGFB induced activation of NFkB leading to increased MDM2 ubiquitin ligase via PI3K. Further, overexpression of wild type p65 NFkB increased MDM2 expression while the NFkB inhibitors NEMO-binding domain (NBD) and Bay11-7082 blocked the activin-induced increase in MDM2. In conclusion, in colon cancer cell migration, activin utilizes NFkB to induce MDM2 activity leading to the degradation of p21 in a PI3K dependent mechanism. This provides new mechanistic knowledge linking activin and NFkB signaling in advanced colon cancer which is applicable to targeted therapeutic interventions.

## INTRODUCTION

Colorectal cancer (CRC) remains one of the most common types of cancer and constitutes a leading cause of cancer death worldwide due to its high incidence and mortality through advanced stage presentation. Although localized forms of CRC can be effectively managed, no curative treatment is currently available for metastatic CRC. At present, targeted therapies for metastatic CRC such as the angiogenesis inhibitor, bevacizumab, or the epidermal growth factor receptor (EGFR) inhibitor, cetuximab, have been used, but with only incremental effects [1–4]. Therefore, treatment of advanced CRC requires ongoing efforts to add new strategies to our armamentarium.

TGFB super family members play an important role in CRC. The signaling pathways for activin and TGFB are frequently disrupted in CRC, however, intact signaling at later stages has been associated with poor prognosis [5]. Early disruption of TGFB-SMAD and downstream cyclin dependent kinase inhibitor p21 signaling can contribute to CRC carcinogenesis [6–8]. We have previously reported that TGFB and activin differentially regulate p21, and that this is associated with advanced disease [6, 7]. However, the precise mechanisms of activin-induced migration are not completely known nor are the link to other inflammatory pathways.

Nuclear Factor kappa B (NFkB) is a pleotropic transcription factor which regulates expression of a number of genes that promotes cell growth, survival and neoplastic transformation in a wide range of tumors [9–11]. In its canonical signaling pathway, transcriptionally competent NFkB is a heterodimer composed of p50 and p65 (RelA) subunits [12, 13]. In unstimulated cells, this heterodimer is sequestered in the cytoplasm by p65 bound IKBα [14]. Upon stimulation, IKBα is phosphorylated by the IKB kinase (IKK) and targeted for degradation via the proteasome pathway. This liberates NFkB and promotes its nuclear translocation where it activates the transcription of target genes that influence cellular proliferation, migration and inflammation. Compared to normal colorectal epithelial cells, cancer cells exhibit aberrant constitutive NFkB activation which is involved in multiple signaling cascades related to carcinogenesis, including survival, invasion and migration of cancer cells [15–17].

Increased expression of MDM2, a RING-figure-containing E3 ubiquitin ligase, is tightly associated with poor prognosis in a variety of tumors, including CRC [18–21]. MDM2 negatively regulates p21 by reducing its stability via proteasome-mediated degradation [22, 23]. The oncogenic action of MDM2 may therefore be associated with negative regulation of p21 and the removal of cell cycle inhibition. Regulation of MDM2 gene expression is dependent on a number of transcription factors including NFkB and the MDM2 gene has been reported to have NFkB binding sites [24–26].

In this current study, we sought to elucidate the mechanisms underlying activin-induced migration in CRC and specifically a potential link between NFkB and activin in metastatic CRC.

## RESULTS

### Activated NFkB and elevated activin are associated and correlate in patients with metastatic CRC

**Figure 1:**
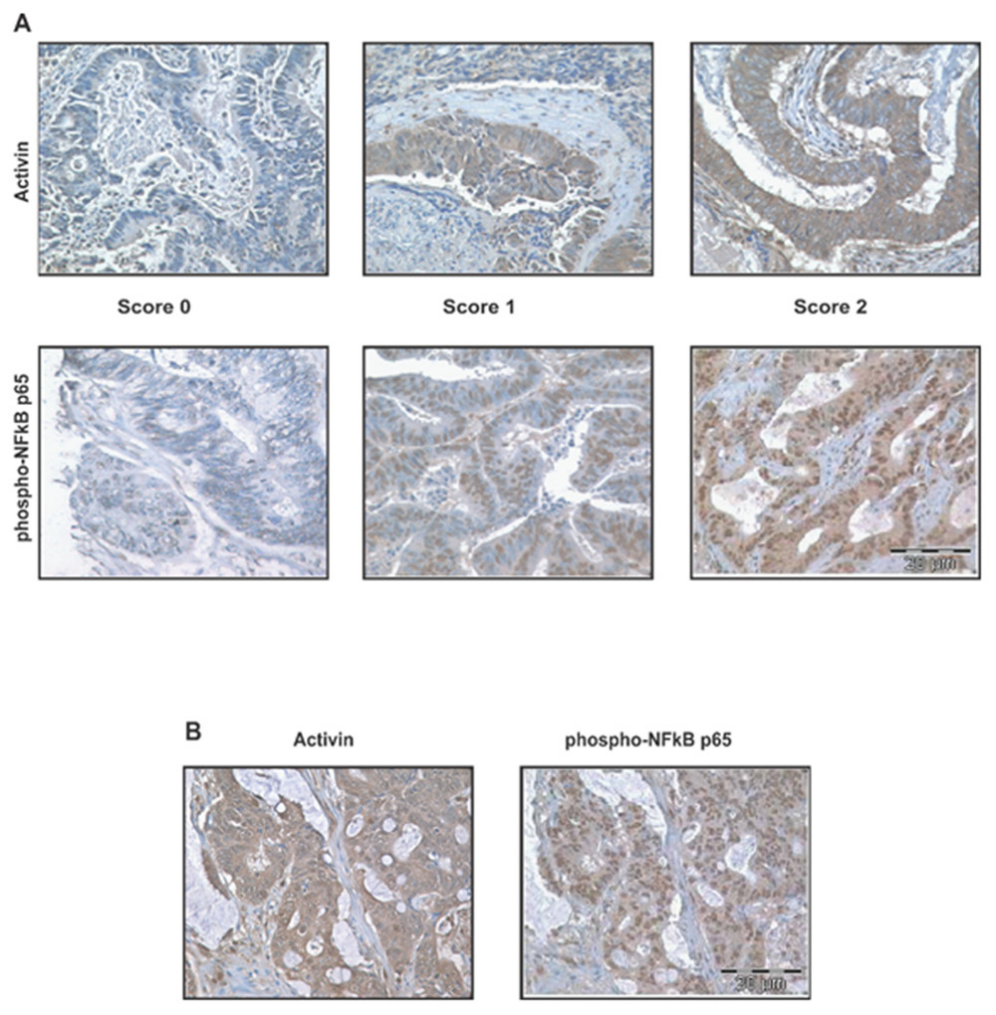
Activin ligand and activated NFkB correlate in colon cancers Slides shown are examples of TMA of 4 tissue array blocks. **(A)** Upper panel shows representative slides of activin staining and lower panel nuclear phospho p65 staining. TMA consisting of colonic adenocarcinomas and adjacent normal colonic mucosa from 131 colorectal cancer patients were scored for non-nuclear activin staining and nuclear NFkB (phospho p65). A score of zero represented weak or negligible staining while 1 equaled moderate staining and 2 equaled strong staining as described in Materials and Methods. **(B)** Staining of a representative, matched colon cancer from the TMA with correlation of high activin ligand and activated NFkB [Kendall's tau_b test (r=0.542)]. Scale represents 20μm.

Constitutive NFkB signaling has been implicated in the development and progression of several cancers, including CRC [11, 27–29] and there is an established link between inflammation and colon cancer via activation of the NFkB pathway [30]. We have previously observed that the inflammatory cytokine activin down regulates p21 and increases migration and invasion of colon cancer cells [6, 7]. Activin is important in inflammation and CRC but a potential cross-regulation between NFkB and activin has not been determined. Here, we first determined whether there was a correlation between activated NFkB and activin ligand expression in tumor tissue from CRC patients. Activation of the NFkB pathway results in translocation of NFkB subunits including phospho-p65 to the nucleus [13]. We employed immunostaining for phospho-p65 and activin ligand expression in a tissue microarray (TMAs) consisting of adenocarcinoma and adjacent normal epithelial mucosa from 131 colorectal cancer patients. The TMA staining was scored independently by two investigators as described in Materials and Methods with representative staining shown in Figure 1A. In tumor tissue more than half of the samples showed strong nuclear phospho-NFkB p65 staining in malignant epithelial cells (Table 1). Activin staining was significantly higher in the cytoplasm of malignant epithelial cells (Figure 1A). High levels of nuclear phospho-p65 and high levels of cytoplasmic activin correlated significantly (n=114, Kendall's tau b =0.542, p<0.001 and Table 1). A representative colon cancer sample with high activin and high phospho-p65 staining is shown in Figure 1B. The expression of phospho-p65 or activin did not correlated with age, gender or race (Table 2), but it did correlate with metastasis (p value for two-tailed fisher's exact test = 0.0001). With this, we then sought to determine a potential mechanistic link between these pathways.

**Table 1:** Correlation between phospho-NFkB p65 and activin in colon cancer tissues

Activin	phospho NFkB p65		
	−	+	++
**−**	13	13	2
**+**	1	22	23
**++**	1	11	28
**N**	15	46	53

Results of TMA staining where levels of nuclear phospho-p65 correlate with activin ligand staining as either weak (-), moderate (+) or strong (++).

**Table 2:** The relationship between NFkB, activin expression and the clinopathological factors in CRC

Clinicopathological factors	NFkB (%)	Activin (%)	Overlap	p-value
**Age**				
≤ 60yrs (n=40)	18 (45%)	14 (35%)	8	
> 60yrs (n=58)	28 (48.28%)	19 (32.76%)	15	0.6293
**Sex**				
male (n=45)	23 (51.11%)	19 (42.22%)	15	
female (n=53)	23 (43.40%)	14 (26.42%)	8	0.0544
**Staging**				
stage I + II (n=40)	9 (22.5%)	12 (30%)	6	
stage III + IV (n=26)	22 (84.61%)	11 (42.30%)	11	0.0206
**Race**				
white (n=45)	24 (53.33%)	14 (31.11%)	8	
nonwhite (n=53)	22 (41.51%)	19 (35.85%)	14	0.3410

The number of patients in each category is followed by the percent. Overlap indicates the number of patients in each category with high dual staining for both nuclear phospho-p65 (NFkB) and activin.

### Activin but not TGFB reduces IKBα protein expression by a PI3K-dependent pathway in colon cancer cells

**Figure 2:**
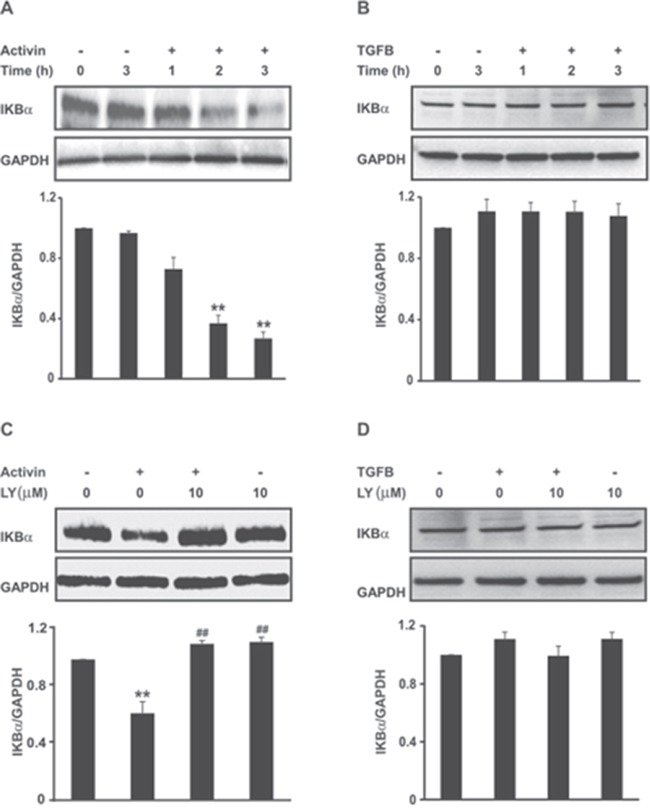
Activin but not TGFB reduces IKBα protein expression in a PI3K-dependent manner **(A** and **B)** FET colon cancer cells were stimulated with activin (25ng/ml) or TGFB (10ng/ml) for the indicated time points followed by immunoblot analysis of total IKBα protein levels (upper panel). **(C** and **D)** Inhibition of PI3K abrogate activin-mediated suppression of IKBα expression in FET colon cancer cells. Cells pre-incubated with the pharmacological PI3K inhibitor LY294002 (10uM) for 30 min were stimulated with activin (25ng/ml) or TGFB (10ng/ml) under serum-free condition for 3hours followed by western blotting. Densitometric analysis for IKBα (IKBα/GAPDH) is shown below each representative blot with ± SD of three independent experiments. **p < 0.01 versus control; ## p < 0.01 versus activin.

We used the fully characterized FET colon cancer cell line [31, 32], which is highly differentiated and expresses fully functional activin and TGFB signaling pathways [7, 33], to assess the activation of NFkB by activin. We first investigated the effect of activin on the expression of the NFkB inhibitor protein, IKBα. IKBα inhibits NFkB activation by cytoplasmic sequestration, while a decrease in IKBα releases NFkB from the cytoplasm to enter the nucleus in an active state. As is evident in Figure 2A, activin markedly decreased the levels of IKBα. In contrast, at this time point, TGFB treatment did not decrease IKBα levels under similar experimental conditions (Figure 2B) alluding to an activin ligand specific effect.

The PI3K pathway plays an important role in cancer and is a critical effector of multiple growth factor receptors. We have recently shown that activin utilizes PI3K signaling to increase colon cancer cell migration [6]. Studies have shown there is cross talk between the oncogenic PI3K and NFkB pathways, which both promote cancer progression [34, 35]. To investigate the involvement of PI3K in regulating NFkB activation by activin, we examined the effects of LY294002 (LY), a potent PI3K inhibitor, on IKBα expression. As observed in Figure 2C, diminished IKBα levels in activin treated cells were restored when PI3K was inhibited by LY. In contrast, we did not observe any effect with LY on IKBα levels in TGFB treated cells, which is consistent with our previous observations that only activin, and not TGFB, activates the PI3K pathway [6, 7].

### Activin but not TGFB promotes PI3K-dependent DNA binding of NFkB in colon cancer cells

**Figure 3:**
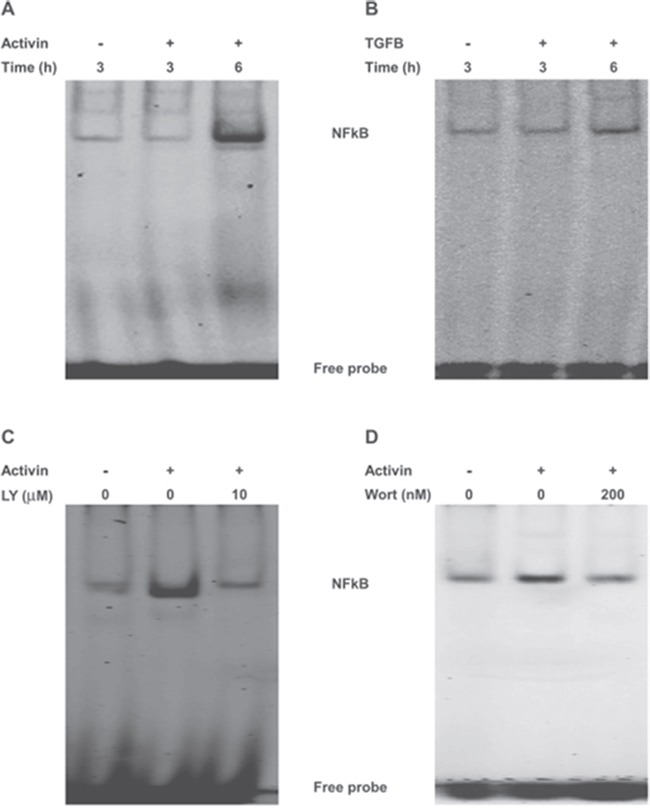
NFkB and PI3K cross-talk in colon cancer cells to affect NFkB DNA binding **(A** and **B)** FET colon cancer cells were treated with activin (25ng/ml) or TGFB (10ng/ml) for the indicated time points under serum free condition. Nuclear extracts were prepared followed by monitoring of DNA-binding activity of NFkB by electrophoretic mobility shift assay (EMSA) using the infrared-labeled consensus NFkB-binding sequence (5′-AGT TGA GGG GAC TTT CCC AGG C-3′; see Materials and Methods). The upper band indicates the induced NFkB while the lower band represents unbound probe, respectively. **(C** and **D)** PI3K inhibition either with LY294002 or wortmanin abolishes activin-induced NFkB activation in FET colon cancer cells. EMSA from nuclear extracts of FET colon cancer cells pretreated either with LY294002 (10uM) or wortmanin (200nM) for 30 min prior to activin stimulation for 6 hours revealed loss of induction following activin with LY or wortmanin treatment. Results shown are representative blots of three independent experiments.

To further investigate the mechanism of PI3K signaling in activin induced suppression of IKBα, we examined the crosstalk between PI3K and NFkB in FET cells, which has been reported in other cell types [36]. Since activin leads to a PI3K-dependent decrease in IKBα expression in FET cells, we examined the crosstalk between activin/PI3K and transcriptionally active NFkB by measuring activin-stimulated changes in NFkB binding to its DNA response element by Electrophoretic Mobility Shift Assay (EMSA) [37]. Binding specificity was confirmed by competition with the unlabeled oligonucleotide for the consensus NFkB binding site or the AP1 consensus binding site which does not bind to NFkB (Supplementary Figure 1A). Treatment of colon cancer cells with activin resulted in increased binding of NFkB to its consensus sequence (Figure 3A). In contrast, TGFB had a minimal effect on the DNA-binding activity of NFkB (Figure 3B). To further confirm these results, we used two inhibitors of NFkB. The activation of NFkB requires prior phosphorylation and degradation of IKBα [13, 16]. The NFkB inhibitor Bay11-7085 inhibits the phosphorylation of IKBα thereby preventing its degradation and the subsequent release of active NFkB [37]. Bay11 treated colon cancer cells displayed a decrease in DNA-binding of NFkB following activin treatment confirming the role of IKBα in this process. We then employed a second NFkB inhibitor that blocks the NFkB essential modifier (NEMO) and IKB kinase (IKK) NEMO-binding domain (NBD) [38]. Wild type NBD peptides (wtNBD) inhibit the induction of NFkB activation without inhibiting basal NFkB activity [38]. Similar to Bay11, wtNBD peptides abrogated activin-induced NFкB DNA binding activity (Supplementary Figure 1B and 1C).

We next examined the requirement of PI3K signaling for constitutive activation of NFkB by EMSA. Pharmacologic inhibition of PI3K either with a reversible inhibitor LY or an irreversible inhibitor wortmanin significantly reduced DNA binding activity of NFkB in activin treated FET colon cancer cells (Figure 3C and 3D). Interestingly, the above effect is identical to that seen following inhibition of NFkB (Supplementary Figures 1B and 1C) and strongly suggests that there is an activin-dependent crosstalk between PI3K and NFkB. To show that the cross talk between NFkB and PI3K is a generalized but not a cell type-specific phenomenon EMSA for NFkB was performed in ACVR2 positive SMAD4 null SW480 cells under identical condition as stated above. Similar to that of FET cells, inhibition of PI3K activation either by LY or wortmanin significantly reduced activin-induced NFkB DNA binding activity as shown in Supplementary Figure 2A and 2B.

### Activin but not TGFB utilizes NFkB to induce MDM2 expression resulting in p21 ubiquitination and degradation in colon cancer cells

Our group has previously shown that one downstream effect of activin-induced PI3K activation is p21 downregulation via ubiquitination [6, 7]. The underlying mechanism of that activin-induced ubiquitination, however, has not been elucidated. We hypothesized that MDM2 is involved in the activin-induced decrease in p21 given that activin increases NFkB activation and MDM2 is an ubiquitin ligase subject to NFkB regulation. In addition, increased expression of MDM2 is associated with many tumor types including melanoma, colon and breast cancers [22, 39]. Therefore, we sought to determine if MDM2 is a downstream effector of PI3K and NFkB signaling in CRC cells exposed to activin. To examine the role of PI3K/AKT signaling in regulating activin-mediated MDM2 expression, FET and SW480 colon cancer cells were pretreated either with LY or wortmanin and exposed to activin for 24h. First, we determined the AKT activation levels in those lysates by immunoblotting with phospho (Ser473) AKT antibody. As expected, PI3K inhibitors LY and wortmanin significantly inhibited activin-induced AKT phosphorylation. Next, to determine whether PI3K inhibition results in decreased MDM2 expression, we analyzed the protein levels of MDM2 in those lysates. As observed in Supplementary Figure 3 and 4, PI3K inhibition decreased activin-induced MDM2 expression in both cell types under similar experimental condition.

**Figure 4:**
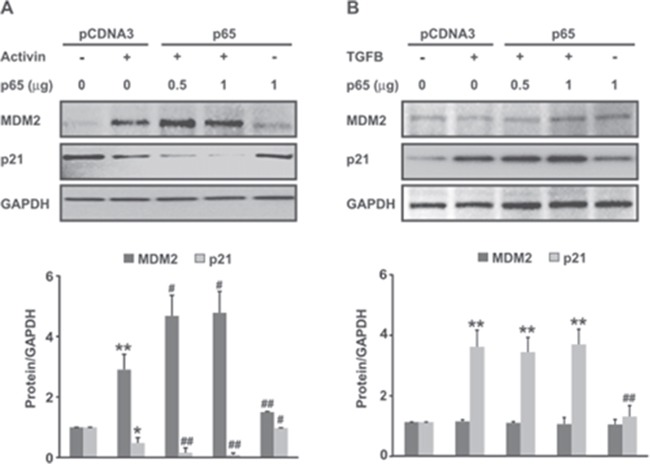
MDM2, an ubiquitin ligase, is induced by Activin and NFkB but not by TGFB **(A** and **B)** 48 hours following transfection of FET colon cancer cells with pCDNA3-p65 or empty vector, cells were treated with activin (25ng/ml) or TGFB (10ng/ml) for an additional 24 hours in serum-free condition followed by immunoblot analysis of MDM2 and its potential downstream target p21. Activin in concert with NFkB overexpression leads to MDM2 overexpression associated with p21 downregulation. Conversely, TGFB does not induce MDM2 expression alone or in combination with over-expressed NFkB. Activin, but not TGFB upregulates MDM2 via NFkB and MDM2 upregulation is associated with p21 downregulation consistent with its ubiquitin ligase activity. Below each immunoblot densitometric analysis of the respective protein (relative to GAPDH) is shown. Results are the representative of three separate experiments.*p< versus pCDNA3; #p <versus activin. */#p < 0.05; **/##p < 0.01 respectively.

**Figure 5:**
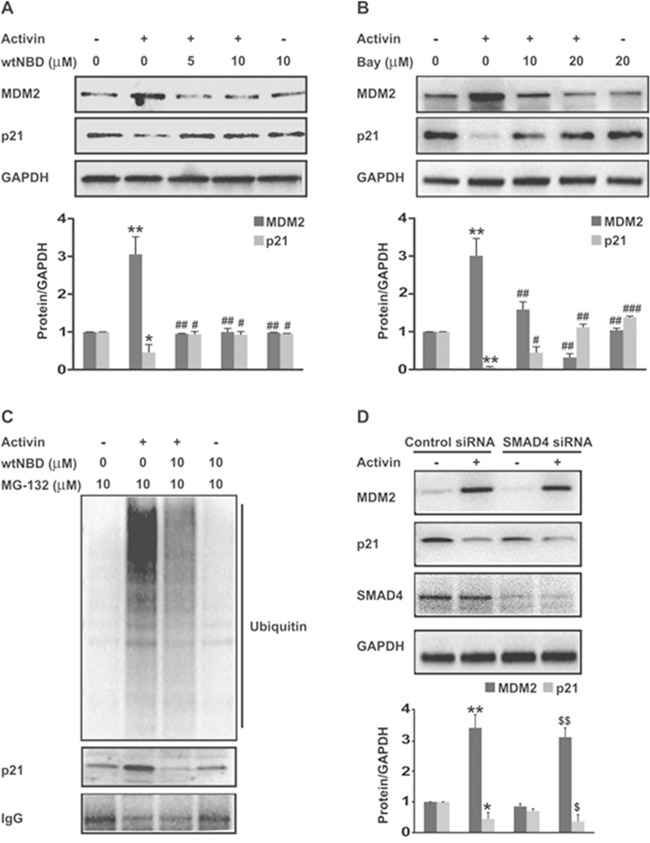
Upregulation of MDM2 expression and p21 ubiquitination by activin via NFkB is SMAD4-independent **(A** and **B)** FET colon cancer cells were pretreated with varying doses of NFkB inhibitors; wild type NEMO-binding domain peptides (wtNBD) or Bay11-7082 for 45 min under serum free condition. This was followed by activin stimulation for 24 hours and immunoblot analysis of MDM2 and p21 protein levels. **(C)** FET colon cancer cells were pretreated with proteasome inhibitor MG132 (10uM) for 30 min prior to activin stimulation for 24 hours. After 24 hours changes in p21 ubiquitination were examined after immunoprecipitation with the anti-p21 antibody followed by immunoblotting with an anti-ubiquitin antibody and reblotting of p21. The ladder of bands represents ubiquitinated p21. Accumulation of higher molecular mass adducts is seen in activin treated cells that was significantly inhibited with wtNBD peptides (10uM). Similar results were obtained in three independent experiments. **(D)** FET colon cancer cells were electroporated with control siRNA or SMAD4 siRNA 48 hours prior to 24 hour activin stimulation and lysates immunoblotted. Knock down efficiency was confirmed by anti-SMAD4 antibody. Activin leads to an increase in MDM2 and decrease in p21 that was not affected by the siRNA-induced loss of SMAD4 expression. Below each immunoblot are densitometric analysis of the respective protein (relative to GAPDH) from at least three independent sets of experiments ± SD. *p indicates versus control; #p indicates versus activin; $p indicates versus SMAD4 siRNA. */#/$ p < 0.05; **/##/$$ p < 0.01; ### p < 0.001 respectively.

Next we investigated the effect of NFkB signaling on activin-induced MDM2 expression. FET colon cancer cells were transfected with a NFkB p65 overexpression plasmid (Supplementary Figure 5). Treatment of colon cancer cells with activin led to an increase in MDM2 protein expression after 24h of stimulation that was further augmented by p65 overexpression. Further, the increase in MDM2 expression correlated with a decrease in p21 protein expression (Figure 4A). In contrast, TGFB did not induce MDM2 protein expression in colon cancer cells nor did it further enhance MDM2 expression in the p65 overexpressing cells (Figure 4B). We also observed a reduction in the levels of activin-induced MDM2 in cells pretreated with wtNBD peptides (Figure 5A), confirming the involvement of NFkB in the activin regulation of MDM2 expression. This reduction in MDM2 was paralleled by an increase in p21 expression (Figure 5A), solidifying the role of MDM2 in activin-induced p21 downregulation. Similarly, Bay11 inhibition of NFkB blocked activin-induced MDM2 expression and simultaneously increased p21 levels (Figure 5B) in a dose-dependent manner.

Next we investigated whether NFkB signaling is responsible for activin-induced p21 ubiquitination and degradation. Proteasomal inhibitors block the binding of ubiquitinated proteins to proteasome, resulting in their accumulation. Therefore FET colon cancer cells were pretreated with MG132 prior to activin stimulation to prevent degradation of p21. As shown in Figure 5C an ubiquitin smear was observed in activin treated cells, indicating that proteasome inhibition allows the accumulation and detection of ubiquitinated p21. Interestingly, the disappearance of the ubiquitin smear is seen following impairment of NFkB activation with wtNBD peptide. This result suggests that activin-induced NFkB signaling accelerates p21 ubiquitination and degradation (Figure 5C). Similarly in activin treated SMAD4 null SW480 colon cancer cells, NFkB inhibition with wtNBD peptide caused reduced expression of MDM2 paralleled with increased p21 protein expression (Supplementary Figure 4C). Together, these results confirm that stimulation of the NFkB pathway by PI3K is required for activin-induced MDM2 expression and p21 ubiquitination and degradation.

### Activin-induced MDM2 expression is SMAD-independent

Loss of SMAD4 protein correlates with a metastatic phenotype, as canonical growth suppressive SMAD signaling is replaced by non-canonical SMAD-independent signaling that promotes proliferation and migration [6–8]. We have previously shown that activin-induced suppression of p21 occurs through a SMAD4 independent non-canonical pathway [7]. Here, we investigated the role of SMAD4 signaling in activin-stimulated changes in MDM2 and p21 expression. siRNA-mediated depletion of SMAD4 did not impair the activin-stimulated induction of MDM2 expression or the resulting decrease in p21 levels in FET cells (Figure 5D). This suggests that activin-induced changes in MDM2 and p21 expression are SMAD4 independent [6, 7].

### Activin-induced transcription of MDM2 is dependent on NFkB

**Figure 6:**
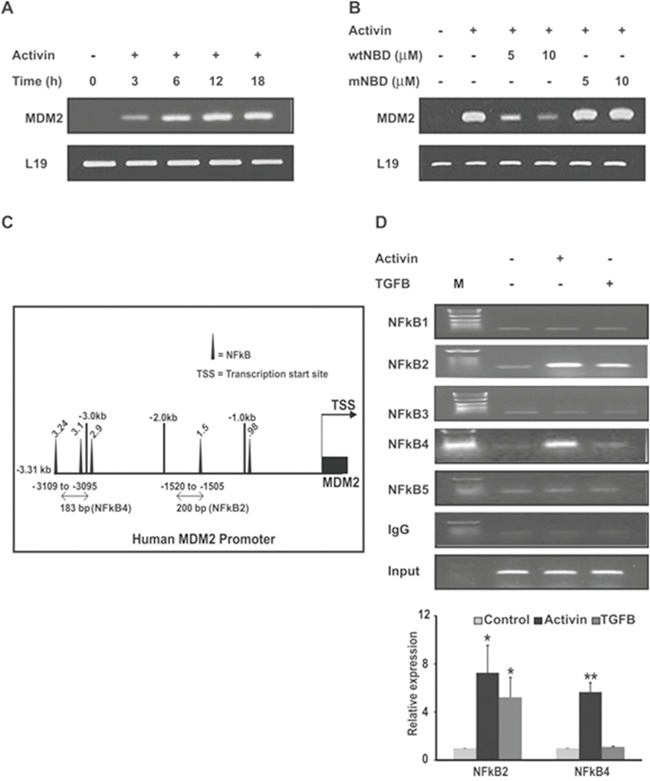
Activin increases MDM2 expression via recruitment of NFkB p65 to the MDM2 promoter **(A)** FET colon cancer cells were stimulated with activin (25ng/ml) for increasing time followed by mRNA analysis of MDM2 by semi-quantitative RT-PCR with detection of L19 as an internal control. **(B)** FET colon cancer cells pretreated with two concentrations of wtNBD peptide or mutant NBD (mNBD) peptide for 45 min were stimulated with activin (25ng/ml) for 6h under serum-free condition followed by monitoring of mRNA expression of MDM2 by qRT-PCR. **(C)** Map of DNA sequence of the human MDM2 promoter region indicates five potential NFkB binding sequences. **(D)** (Upper panel). FET colon cancer cells were treated with activin (25ng/ml) or TGFB (10ng/ml) for 3h under serum-free conditions and then subjected to ChIP analysis by immunoprecipitation of the chromatin fragments with anti p65 or non-specific IgG antibodies. ChIP assay by semi-quantitative PCR show that in the presence of activin treatment p65 binds to two regions in the MDM2 promoter: base pairs -1505 to -1520 and -3095 to -3109 from the transcription start site (TSS) while p65 binds to only one region: base pair -1505 to -1520 in response to TGFB treatment. Immunoprecipitation with non-specific IgG followed by PCR showed almost undetectable bands in semi-quantitative PCR and the total fragmented DNA showed uniform signal, indicating uniformity and specificity of the results. **(D)** (Lower panel). The same reactions for two transcripts NFkB2 and NFkB4 were performed and analyzed by Real-time PCR system. Data is presented as relative expression of p65 signal in treated samples versus the untreated anti p65 antibody using the comparative CT method. The bars presented the expression data for two of the transcripts NFkB2 and NFkB4. M; DNA ladder. All the experiments were repeated at least three times under same conditions. *p indicates versus control. *p < 0.05; **/p < 0.01 respectively.

We next examined the activin-stimulated expression of MDM2 mRNA. As shown in Figure 6A activin induces MDM2 transcription in colon cancer cells. Next, we examined if the activin-induced transcriptional increase of MDM2 requires NFkB by inhibiting NFkB activation with the wtNBD peptides. Interestingly, we found that pretreatment of colon cancer cells with wtNBD peptides reduced the activin-induced increase in MDM2 mRNA expression in a dose dependent manner (Figure 6B). Mutated NBD peptides (mNBD), however, failed to inhibit activin-induced MDM2 mRNA expression. Together, these findings suggest that NFkB is required for activin-mediated upregulation of MDM2 expression.

Previous reports [24] identified binding sites for the NFkB p50/RelA subunits in the MDM2 P1 promoter region. Here we confirmed the p65 (RelA) binding site in the human MDM2 promoter by using the transcription factor binding site search software tool MatInspector. We confirmed five p65 consensus binding sites upstream of the transcriptional start site depicted in Figure 6C. By chromatin immunoprecipitation (ChIP) analysis, we monitored the recruitment of p65 to the MDM2 promoter. Our ChIP assays demonstrated an activin-induced increase in p65 binding to two p65 binding sites in regions -1505 to-1520 and -3095 to -3109 of the human MDM2 promoter (Figure 6D, upper panel). In contrast, p65 only bound to one NFkB site (a 200 bp fragment from -1505 to-1520) in the MDM2 promoter from TGFB-treated cells. To confirm the results obtained by ChIP, we performed quantitative analysis of ChIP transcription level by real-time RT-PCR (Figure 6D lower panel). The two transcripts found to be upregulated by ChIP assay in response to activin stimulation also exhibited increased p65 dependent expression of MDM2 promoter by real-time RT-PCR analysis. In contrast, only one transcript NFkB2 is found to be upregulated by TGFB stimulation. Similar to ChIP assay result, no detectable levels of other transcripts were observed by quantitative real-time PCR (data not shown). Taken together, these data indicate that activin specifically induces the recruitment of p65 to two NFkB binding sites in the MDM2 promoter.

### Inhibition of NFkB leads to a decrease in migration and survival in response to activin stimulation in colon cancer cells

**Figure 7:**
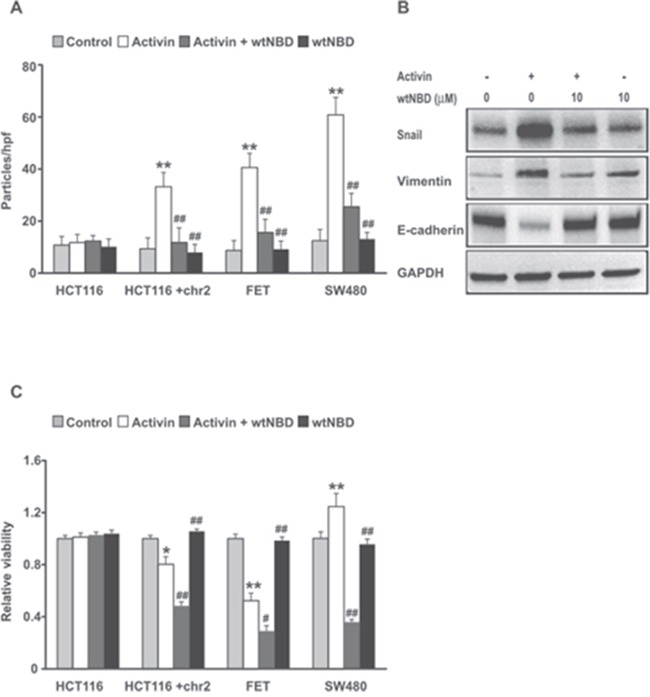
NFkB inhibition suppresses activin-induced migration, epithelial mesenchymal transition and enhances activin-induced loss of cell number in colon cancer cell lines **(A)** A spectrum of colon cancer cells lines including HCT116 lacking the ACVR2 receptor, HCT116+chr2 supplemented with the ACVR2 receptor, ACVR2 positive FET cells and SMAD4 null ACVR2 positive SW480 cell lines were treated as follows: Light gray bars=vehicle control; White bars = 25 ng/ml activin for 6 hours; Dark Gray bars = combination treated cells were pre-incubated with wtNBD peptide (10uM) for 45 min and then stimulated with 25ng/ml activin for 6h under serum free condition and Black Bars = treatment of wtNBD peptide (10uM) alone. The cells were analyzed by transwell migration assay and data are expressed as number of particles per high powered field (hpf) as described in Material and Methods. Results are mean SD of three different experiments. **(B)** Inhibition of NFkB reduces activin-induced upregulation of EMT markers in FET colon cancer cells. The cells were pretreated with wtNBD peptide (10uM) for 45min prior to activin stimulation (25ng/ml) for 72h under serum free condition. Activin treatment induces an increase in expression of mesenchymal markers, vimentin and Snail followed by a decrease in epithelial marker E-Cadherin as examined by western blotting. The above effects were reversed following NFkB inhibition under similar context of activin treatment. The experiments were repeated three times and similar results were obtained. GAPDH was used as a loading control. **(C)** HCT116 and HCT116+chr2, FET and SW480 colon cancer cell lines were treated as indicated above. Relative viability was assessed by MTT assay as described in Materials and Methods. Data were normalized to control. Results are mean SD of three independent experiments. *p indicates versus control; #p indicates versus activin. */#p < 0.05; **/##p < 0.01 respectively.

We have previously reported that activin stimulates migration of CRC cells via non-canonical pathways independent of SMAD4 activation [6]. NFkB activation occurs frequently in colitis-associated CRC and contributes to cancer progression [40]. To ask if NFkB activation plays an important role in activin-induced migration, we inhibited NFkB activation and monitored changes in migration in a panel of colon cancer cell lines including the HCT116 colon cancer cell line model system. The parent line, HCT116, does not express the activin receptor, while its derivative, the HCT116+2 cells, are supplemented with human chromosome 2 which restores expression and function of activin receptor, ACVR2 [41]. The FET colon cancer cells have functional activin signaling and the SW480 colon cancer cells have functional ACVR2, but lack SMAD4. As expected, in the absence of the activin receptor (HCT116) there is no activin induction of cell migration, nor is there any change in cell migration when the NFkB activation is inhibited with the wtNBD peptide (Figure 7A). Restoration of the activin receptor enabled activin-induced cell migration that was suppressed by inhibition of NFkB. This establishes a functional role for NFkB in activin-induced migration. Similarly, in ACVR2 wild type FET cells, activin caused an increase in migration that was attenuated by NFkB inhibition with wtNBD peptides. The lack of SMAD4 in the SW480 cells increases the ability of activin to stimulate migration in these cells. This confirms earlier reports which suggest that activin-induced migration is via the non-canonical (SMAD4-independent) pathway. Taken together these results indicate that activin-induced migration requires NFkB and further this requires a functional ACVR2, but not SMAD4.

Epithelial to mesenchymal transition (EMT) generally precedes increased migration of cancer cells during which epithelial marker E-cadherin is downregulated with a concomitant upregulation of mesenchymal markers, snail and vimentin. To test if activin treatment changes expression of EMT proteins FET colon cancer cells were treated with activin for 72h. Immunoblot analysis of lysates from activin-treated cells (Figure 7B) showed increased expression of snail and vimentin followed by a decrease in E-cadherin expression. These events were reversed by wtNBD peptide indicating a requirement for NFkB.

Since NFkB activation influences both migration and cell viability by inducing genes involved in matrix degradation as well as genes involved in survival function [42, 43], we examined the effect of NFkB inhibition on cell viability with respect to activin stimulation. We have previously reported that activin decreases cell viability in colon cancer cells [7, 41] through a SMAD4-dependent pathway (canonical signaling). Here we assessed metabolic activity via MTT assay as an indirect measure of cell viability. This assay was performed to measure cell viability in response to activin stimulation with and without wtNBD peptide mediated inhibition of activated NFkB. We observed no change in cell viability with any treatments in the ACVR2 null HTC116 cells (Figure 7C). Activin reduced cell viability in ACVR2+ FET cells and ACVR2-restored HCT116+chr2 colon cancer cells (Figure 7C). This decrease was further enhanced by wtNBD peptides. Interestingly, in the SMAD4-null SW480 cells, activin treatment increases cellular proliferation reflecting the unopposed activation of NFkB. As expected, this increase in activity is sensitive to NFkB inhibition by wtNBD peptides suggesting that inhibition of NFkB activation may be an effective therapeutic approach for colon cancers regardless of SMAD4 status.

In summary, we show that in colon cancer, activin activates NFkB via PI3K increasing migration via p21 regulation through the MDM2 ubiquitin ligase. Taken together, the results presented here further support a link between inflammation and advanced cancer and pave the way for future studies elucidating targets, risk stratification and prevention.

## DISCUSSION

Activin is an important cytokine both in cancer [41, 44] and in inflammation [45]. We have previously shown that activin signaling is commonly disrupted in colon cancer to affect loss of growth suppression, but that intact activin signaling is prometastatic at later stages [6, 7, 41, 46]. In addition, we have observed that activin utilizes PI3K via non-SMAD signaling to increase p21 degradation in colon cancer cell migration [6], but the greater context of these findings specifically as they pertain to an inflammatory environment needs to be established.

**Figure 8:**
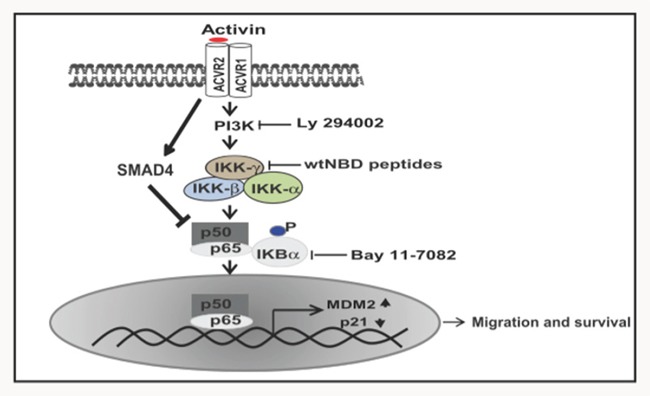
Schematic diagram of proposed activin pathway signaling through NFkB activation In response to activin stimulation, IKBα proteins are degraded in a PI3 kinase-dependent manner. This results in NFkB translocation to the nucleus leading to transcription of its target gene MDM2 and subsequent downregulation of p21. Activin signaling through NFkB results in increased cellular migration and that decreased survival of colon cancer cells is due to suppression of NFkB signaling by SMAD4.

NFkB is critical to the inflammatory response and previous work has established that activin is an inflammatory cytokines that can activate NFkB [47]. Others have shown that epithelial activation of NFkB is crucial for the development of colitis-associated colorectal cancer [30]. However, until now, the cross-talk between activin and NFkB in the colorectal epithelium or this pathway's relationship to colorectal cancer has not been investigated. Here we have established a direct link between activin ligand expression and activated NFkB via nuclear localization of the phosphorylated p65 NFkB subunit in colon cancer patients. Further, this link correlated with more advanced disease. We determined that activin mediates its effects by stimulating the PI3K-dependent p65 NFkB resulting in increased transcription of MDM2 and degradation of p21 (Figure 8). By contrast, this NFkB mediated effect did not occur in TGFB stimulated cells, which underscores our previous findings that activin and TGFB, while sharing downstream SMAD signaling, diverge in regards to oncogenic non-SMAD signaling [6]. These results indicate when SMAD4 signaling is intact, activin has an anti-proliferative effect as SMAD4 is reported to increase SMAD7 transcription leading to an increase in NFkB inhibitor protein IKBα [48–50]. Therefore suggesting intact SMAD4 signaling is dominant over NFkB that in turn counterbalance the proliferative actions of activin-induced NFkB.

Although others have observed activation of NFkB by TGFB at early time points using *in vitro* models of CRC, this activation was transient and of short duration returning to baseline within 60 minutes [33]. In contrast, our data demonstrate prolonged activation of NFkB by activin *in vitro* further confirmed by strong *in vivo* correlation of nuclear phospho-p65 and activin. This further solidifies the likely distinct role of activin in the context of pro-oncogenic epithelial signaling in CRC.

The cross-talk between the activin and NFkB signaling pathways may also be a central component of the context specific dual pro- and anti-tumorigenic actions described for activin. In models of breast cancer, activin has been reported to be tumor protective as neutralizing activin actions with either small molecule inhibitors or siRNA approaches leads to increased proliferation and cellular migration [51, 52]. We have previously reported in models of colon cancer that activin inhibition of cell proliferation is SMAD dependent. In contrast activin stimulation of oncogenic actions, as measured by increased epithelial to mesenchymal transition (EMT) and increased cellular migration and invasion, are SMAD independent and require activation of PI3K [6, 7]. In this current report, activin's pro-oncogenic actions appear to be not only mediated by NFkB as one of many effectors, but unexpectedly NFkB appears to be central to the detrimental aspects of activin signaling in CRC leading to cellular proliferation in the context of SMAD4-independent signaling.

The current literature indicates that inhibition of NFkB is a promising therapeutic approach, and several candidate therapies for clinical inhibition of NFkB are progressing to clinical trial [53]. However, identification of which patient populations would most benefit from this therapeutic approach is a crucial step for successful application of precision medicine and is lacking in CRC. Our current data support the hypothesis that CRC patients with high tumor expression of activin ligand may benefit from clinical inhibition of NFkB.

## MATERIALS AND METHODS

### Reagents

NBD peptides (>99% pure) were synthesized in the custom peptide synthesis facility of Peptide 2.0 (Chantilly, VA). The peptide consists of two domains: an antennapedia homeodomain (lower case) that confers cell permeability and the region of IKKβ (upper case) which is the NEMO binding domain (NBD). Wild type (wt) peptide is converted to mutated (m) NBD peptide by W to A mutation. The sequence of wtNBD peptide is drqikiwfqnrrmkwkkLDWSWL and mNBD peptide is drqikiwfqnrrmkwkkLDASAL respectively. NFkB inhibitor Bay 11-7082 is from sigma (B5556).

### Tissue microarray (TMA)

The human colorectal carcinoma tissue array study was approved by the UIC Institutional Review Board. Briefly, formalin fixed paraffin embedded patient derived colon tissues were obtained from patients diagnosed with colorectal carcinoma at the University of Illinois Medical Center. The quality and structural integrity of the tissue blocks were appraised and evaluated before inclusion into the study. A total of one hundred thirty one (131) colorectal resection specimens obtained from patients diagnosed with colorectal carcinoma were employed for the construction of the progression tissue array utilized in the study. The tissue array was designed and constructed according to published methods [54]. A total of four tissue array recipient blocks were created comprising of duplicate 0.6 mm diameter tissue cores representing normal colonic mucosa, hyperplastic colonic mucosa, dysplastic colonic mucosa, and colonic adenocarcinoma. Four micron thick tissue array sections were placed on positively charged glass slides for immunohistochemical studies.

### Immunohistochemistry

Formalin fixed and paraffin embedded TMAs were de-paraffinized in xylene and then rehydrated in descending ethanol series. TMA slides were then immersed in 3% hydrogen peroxide for 15 min to block endogenous peroxidase activity and then blocked in 3% BSA for 60 min to block nonspecific binding sites. Immunostaining was performed at 4°C overnight with a rabbit polyclonal NFkB p65 (phospho S536) antibody (1:500 dilution; Abcam, CA, USA) or mouse monocolonal Activin antibody (1:100 dilution; Ansh Labs, TX, USA). Following primary antibody incubation the sections were incubated in HRP-conjugated rabbit or mouse secondary antibody and treated with DAB substrate for staining (Dako, CA, USA). Slides were counterstained with hematoxylin and observed under light microscope. For negative controls, a TMA slide was incubated under similar conditions without the primary antibodies.

### Scoring of IHC staining result

The colon cancer specimens on the TMA slides were examined and scored using a two-headed microscope. A staining index for each tissue core was obtained based on published methods [55]. The immuno-histochemical staining intensity of the nuclei or cytoplasm was assessed using a three tier grading system (weak or no staining = 0, moderate staining = 1, strong staining = 2) and the extent of stained nuclear positive cells (0-50%=1, 50-100%=2). The physical characteristics of the tumor, background pathology, and patient demographic information were recorded. The final immunoreactive score of phospho p65 was determined by multiplying the intensity scores with the extent of positivity scores for nuclei. For activin intensity of cytoplasmic staining was considered. Slides were scored in a blinded fashion by two investigators. Both investigators had to be in agreement for a tumor to be called negative.

### Colon cancer cell lines

SW480, HCT116 and HCT116+2 CRC cell lines were acquired from ATCC and the FET CRC cell line was a generous gift from Michael Brattain, University of Nebraska, Omaha, NE. All cell lines were cultured in a 5% CO_2_ chamber in 10% FBS and 1% PenStrep containing media. All cells were serum starved for 24 hours prior to treatment. To limit variability between experiments, cells were cultured to a maximum of 30 passages to minimize mutations in the cell lines. In addition, cell cultures under identical conditions of treatment were done in parallel to validate our findings. The cells were authenticated once a year and validated by 9 STR (short tandem repeat) profiling using Cell Check 9Plus and tested for mycoplasma (both IDEXX, Columbia, MO, USA) [6].

### Western blot analysis

Cell lysates were prepared for western blot analysis as previously described [6]. Blots were probed with IKBα (1:250 dilution), SMAD4 (1:100 dilution), p21 (1:250 dilution) (Santa Cruz Biotechnology, TX, USA), MDM2 (1:200 dilution) (R&D Systems, MN), and p65 (1:1000 dilution) (Cell Signaling Technology, MA, USA); exposed to HRP-conjugated secondary antibodies, and imaged using enhanced chemiluminescence. Densitometric analysis of immunoblots for respective proteins (IKBα, SMAD4, MDM2, p21 and p65) was done by using image J software. All the experiments were repeated at least three times under same conditions.

### Electrophoretic mobility shift assay (EMSA)

Nuclear extracts were prepared, and electrophoretic mobility shift assay (EMSA) was performed as described previously [56–58] with some modifications. Briefly, IRDye infrared dye end-labeled oligonucleotides containing the consensus binding sequence for NFkB (5’- AGT TGA GGG GAC TTT CCC AGG C-3”) were purchased from Licor Biosciences. Six micrograms of nuclear extract was incubated with binding buffer and with infrared-labeled probe for 20 min. In the competition experiments, unlabeled oligonucleotide probe AP1 or NFkB was incubated with nuclear extract and binding buffer for 20 min. Subsequently, samples were separated on a 6% polyacrylamide gel in 0.25x TBE buffer (Tris borate-EDTA) and analyzed by the Odyssey Infrared Imaging System (LI-COR Biosciences).

### Transfection

FET colon cancer cells were transfected with either 0.5ug or 1ug of plasmid DNA encoding p65 using AMAXA Nucleofector (Lonza, Basel, Switzerland) in 12-well plates at a density of 1×10^6^ according to the manufacturer's instruction. After 48 hours of transfection, cells were treated with activin (Ansh Labs, TX, USA) or hTGFβ (R&D Systems, MN) for an additional 24h followed by lysate preparation for western blot analysis. Specific siRNA for SMAD4 (Ambion, TX, USA) was transiently delivered at a final concentration of 10nM via electroporation. Transfection efficiency was confirmed by fluorescent microscope using the pmaxGFP control vector. Forty-eight hours post transfection, colon cancer cells were pretreated with wtNBD peptides for 45 min followed by activin stimulation for an additional 24h. After 24h cell lysates were prepared for western blot analysis.

### Ubiquitination assay

Ubiquitination assay was performed as previously described [7]. Briefly, 10uM of proteasomal inhibitor MG132 (Calbiochem) was used to block the degradation of p21 for 30min prior to activin stimulation for 24h. After 24h, p21 was immunoprecipitated with anti p21 antibody (sc-469, Santa Cruz Biotechnology) and western blot was performed. For ubiquitination assay, after transferring the proteins to nitrocellulose membrane the membrane was boiled for 15 min prior to blocking and probed with antibodies against ubiquitin (1:1000; sc-8017, Santa Cruz) and p21 respectively.

### Semiquantitative reverse transcriptase- PCR (RT-PCR)

Total RNA was isolated from FET colon cancer cell lines using RNA-Easy Qiagen kit following the manufacturer's protocol as previously described [56]. To remove any contaminating genomic DNA, total RNA was digested with DNAse. Semiquantitative RT-PCR was performed using SuperScript^TM^ III First Strand Synthesis SuperMix and oligo (dT) 20 primers (Invitrogen) in a 20ul reaction mixture. The resulting cDNA was diluted (1:10), and amplified using Taq DNA polymerase (PCR master mix, Promega) with human specific primers (Supplementary Table 1). Amplified products were electrophoresed on a 1.8% agarose gels and visualized by SYBR Safe DNA gel stain (Invitrogen, MA, USA). Message for L19 was used to ascertain that an equivalent amount of cDNA was synthesized from different samples. Semiquantitative RT-PCR was performed at least three times from three different RNA extractions for FET cell line under identical conditions of treatment.

### Quantitative real time PCR analysis

Diluted cDNA templates (1:10) were amplified using SYBR Green Master Mix in triplicates and analyzed by the Bio-Rad CFX 96 Real-time PCR detection system using the following program 95°C for 3 min followed by 50 cycles at 95°C for 10s and 55°C for 30s. Data were processed by the Bio-Rad CFX manager software 3.1. Real time PCR results were expressed and analyzed relative to CT (threshold cycle) values. CT value was normalized to untreated mRNA of each sample. Primers used are listed in the Supplementary Table 1.

### Chromatin immunoprecipitation assay (ChIP)

Briefly, 2 × 10^6^ FET colon cancer cells were stimulated with activin or TGFβ for 3 hours and ChIP assay was performed as described previously [59]. After stimulation, cells were fixed by adding formaldehyde (1% final concentration), and cross-linked adducts were resuspended and sonicated, resulting in an average chromatin fragment size of 400 bp. ChIP was performed on the cell lysate by overnight incubation at 4°C with 2 μg of Abs against either p65 or normal IgG as a non-specific control (Santa Cruz Biotechnology, TX, USA). This is followed by incubation with protein G-agarose (Santa Cruz Biotechnology, TX, and USA) for 2h. The beads were washed and incubated with elution buffer. To reverse the cross-linking and purify the DNA, precipitates were incubated in a 65 °C and digested with proteinase K. DNA samples were then purified and precipitated, and precipitates were washed with 75% ethanol, air-dried, and resuspended in Tris-EDTA buffer. The primers used to amplify fragments flanking proximal as well as distal NFkB binding elements in the human MDM2 promoter are listed in Supplementary Table 1.

### Migration assay

Transwell 12 well plates (8μm pores, Corning, NY, USA) were coated with fibronectin (Sigma) to facilitate migration were seeded with 5 × 10^4^ colon cancer cells per well. Following an incubation period of 6h, non-migrated cells at the membrane top were removed and the cells that have migrated through the membrane were fixed with 4% PFA followed by DAPI (nuclear stain) staining. Images were captured by fluorescent microscopy and the number of migrated cells per well was determined by counting the cells at the center of each well using image J software to digitized images, reduce background and quantify number of particles detected. Data are expressed as number of particles counted per high powered field (hpf).

### Cell metabolic activity

Mitochondrial activity was measured with the 3-(4, 5-dimethythiazol-2-yl)-2-5-diphenyltetrazolium bromide (MTT) assay (cell counting kit-8, Dojindo Molecular Technologies, MD, USA) as previously described [41]. This assay was used as an indirect measure of cell viability and relative viability was calculated in comparison to untreated controls. The cells were grown on 24-well culture plates with 500ul of medium pretreated with wild type NBD peptide followed by activin treatment for 24h. At the end of the treatment period 10% of the MTT solution was added to each well and incubated for 1h.

### Statistics

All data are expressed as means ± SD from at least three independent experiments. Statistical analyses for differences were performed via Students's t test when two groups are analyzed and ANOVA test with Tukey's post test was used when more than two groups were analyzed. For correlations analysis significance was determined using the Kendall's tau b test. Fisher's exact test was used to analyze the association between the two variables. The criterion for statistical significance was p=0.05.

## SUPPLEMENTARY MATERIALS FIGURES AND TABLES


