# Supplementary material for: NFkB is essential for activin-induced colorectal cancer migration via upregulation of PI3K-MDM2 pathway
Source: Oncotarget. 2017 Mar 18;8(23):37377–93. doi: 10.18632/oncotarget.16343 (PMC5514916; doi:10.18632/oncotarget.16343)
Supplement: Supplementary file 1 [file oncotarget-08-37377-s001.pdf]

NFkB is essential for activin-induced colorectal cancer migration via upregulation of PI3K-MDM2 pathway

SUPPLEMENTARY FIGURES AND TABLE

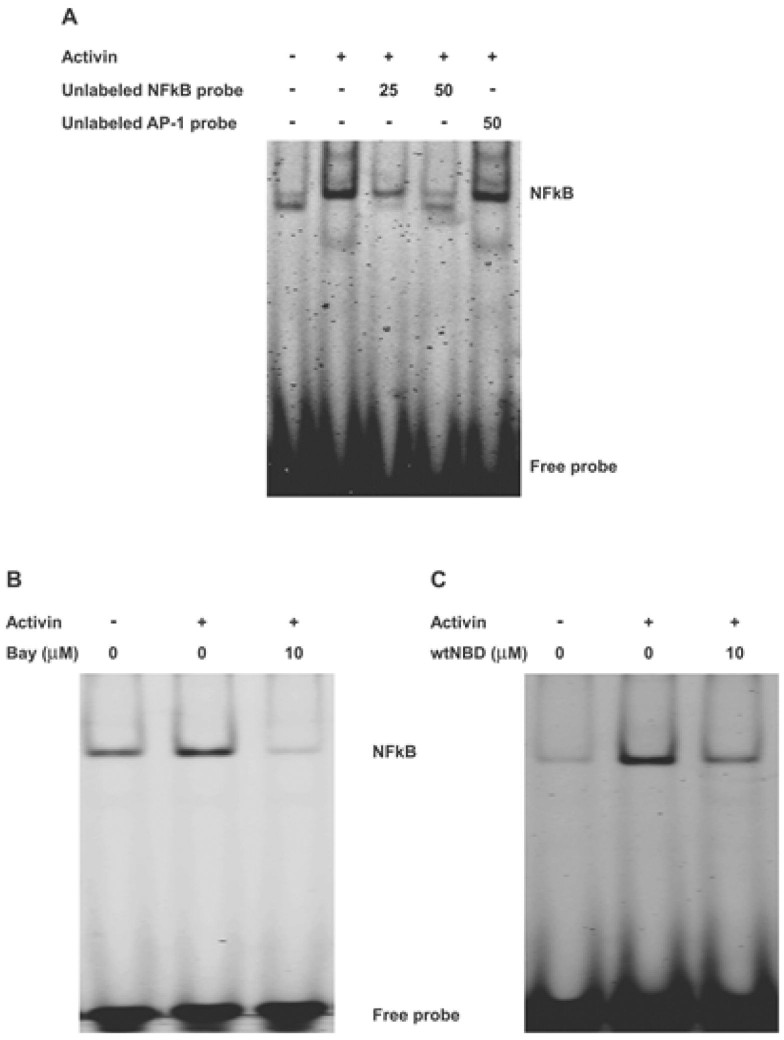

Supplementary Figure 1: Specificity of active NFkB binding in FET colon cancer cells in response to activin stimulation. **(A)** To test for specificity of binding, the binding reaction with nuclear extract was incubated with 25- or 50-fold excess unlabeled NFkB or an unrelated AP1 probe as indicated and EMSA for NFkB was performed as described in Materials and Methods. **(B** and **C)** inhibitors wtNBD peptides and Bay 11-7082 inhibit NFkB activation in activin treated cells. FET colon cancer cells were treated with inhibitors of NFkB activation (either Bay 11-7082 or wtNBD peptides) for 45min prior to 6h activin stimulation. Nuclear extracts were prepared and subjected to EMSA for NFkB. Decrease of activin-induced NFkB by NFkB inhibitors verifies activin treatment leads of increased activity of NFkB. Results are the representative of three separate experiments.

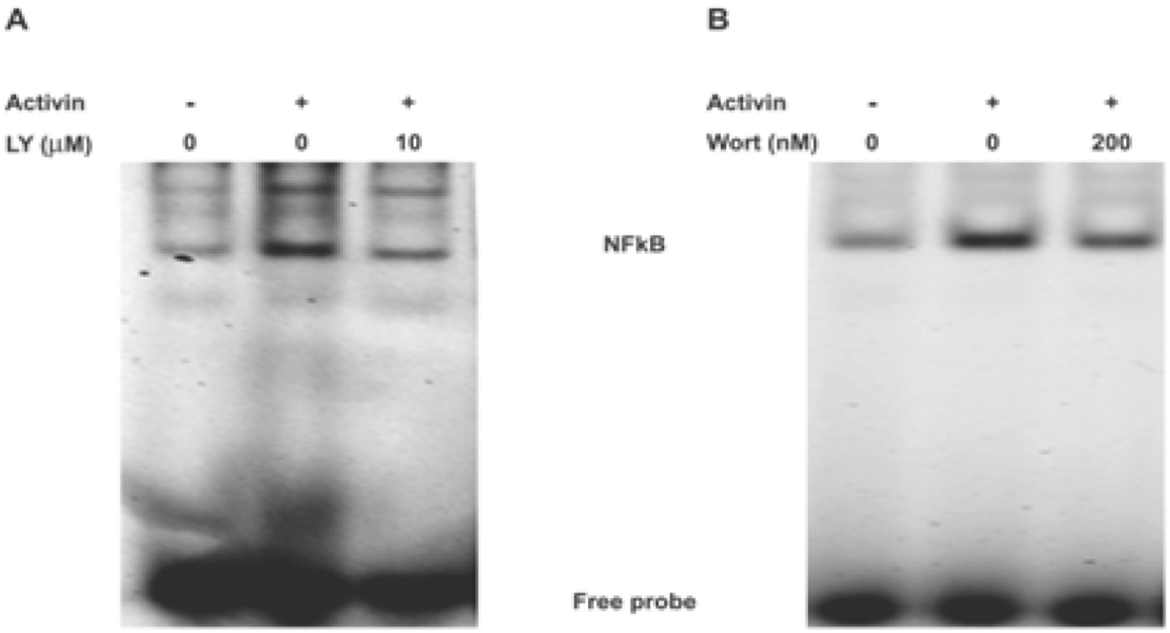

Supplementary Figure 2: Inhibition of PI3K reduces NFkB DNA binding activity. (**A** and **B**) SW480 colon cancer cells were pretreated with LY294002 (10uM) or wortmanin (200nM) for 30 min prior to activin (25ng/ml) stimulation for 6h under serum free condition. After 6h, nuclear extracts were prepared and samples were used in EMSA with oligonucleotides containing elements for NFkB as described in materials and method section. Positions of the NFkB bands and free probe are indicated in the fig. Results shown are representative blots of three independent experiments.

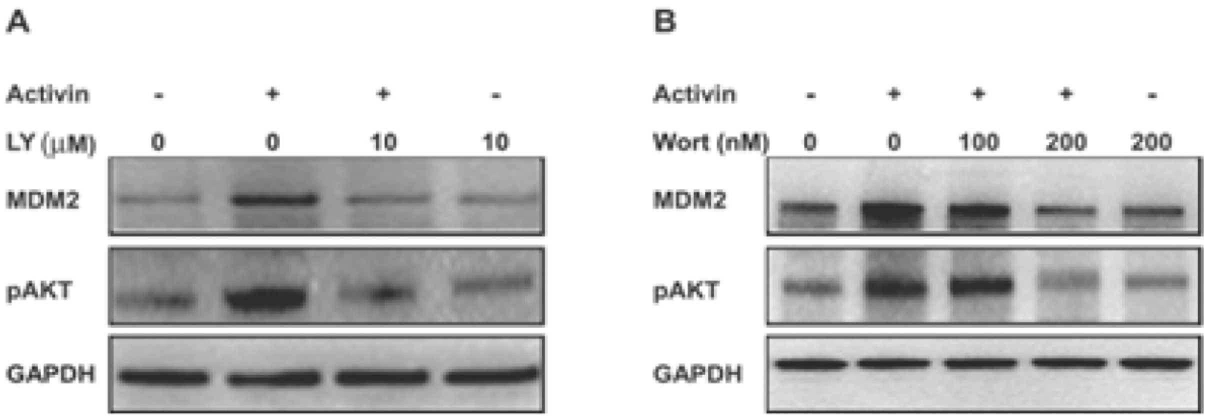

Supplementary Figure 3: The effect of PI3K inhibitors on activin-induced MDM2 expression. (**A** and **B**) FET colon cancer cells were treated either with LY294002 (10uM) or different concentrations of wortmanin as indicated for 30 min followed by activin stimulation for 24h. Following incubation, cell lysates were subjected to western blot analysis for MDM2. Activin-induced upregulation of MDM2 expression was abrogated after PI3K inhibition implicating the involvement PI3K signaling. PI3K inhibitor efficiency is shown by reduced levels of pAkt (Ser473). GAPDH is used as a loading control. Results shown are representative blots of three independent experiments.

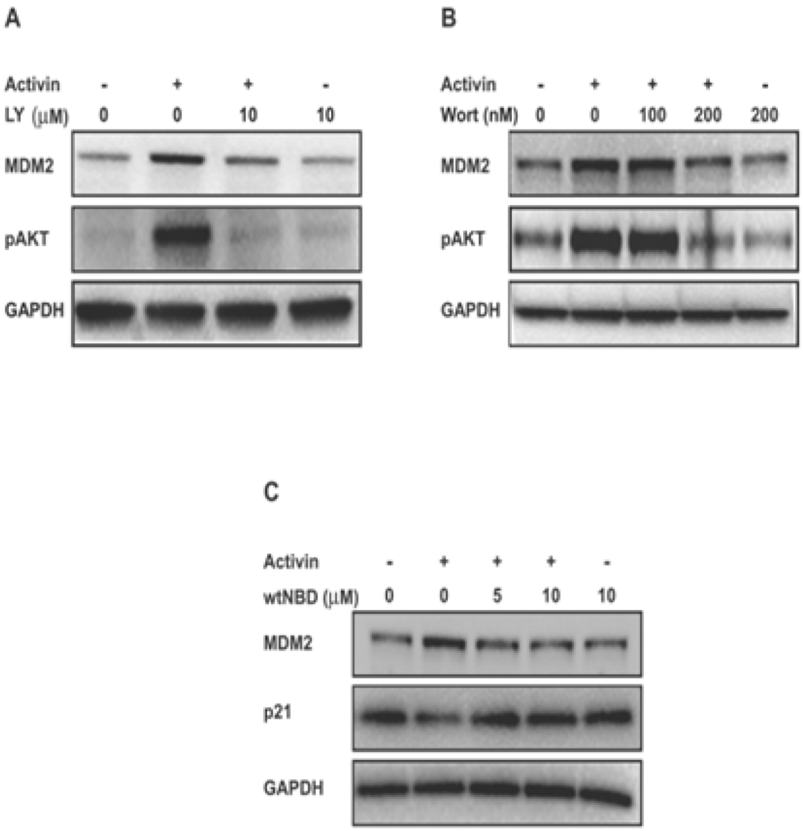

Supplementary Figure 4: Activin utilizes PI3K-NFkB signaling to induce MDM2 expression in SW480 colon cancer cells. (**A** and **B**) Cells were pretreated with LY294002 (10uM) and different doses of wortmanin as indicated above for 30 min followed by activin stimulation for 24h. Lysates were immunoblotted for MDM2. **(C)** Cells were pretreated with different concentrations of wtNBD peptide as indicated for 45 min prior to activin stimulation for 24h followed by immunoblot analysis for MDM2 and p21. Activin-induced MDM2 expression is decreased in the presence of PI3K and NFkB inhibitors as shown by western blot analysis for MDM2. Further, immunoblot analysis of p21 (C) from the same lysates showed that activin-induced MDM2 upregulation is associated with p21 downregulation. Results shown are representative blots of three independent experiments.

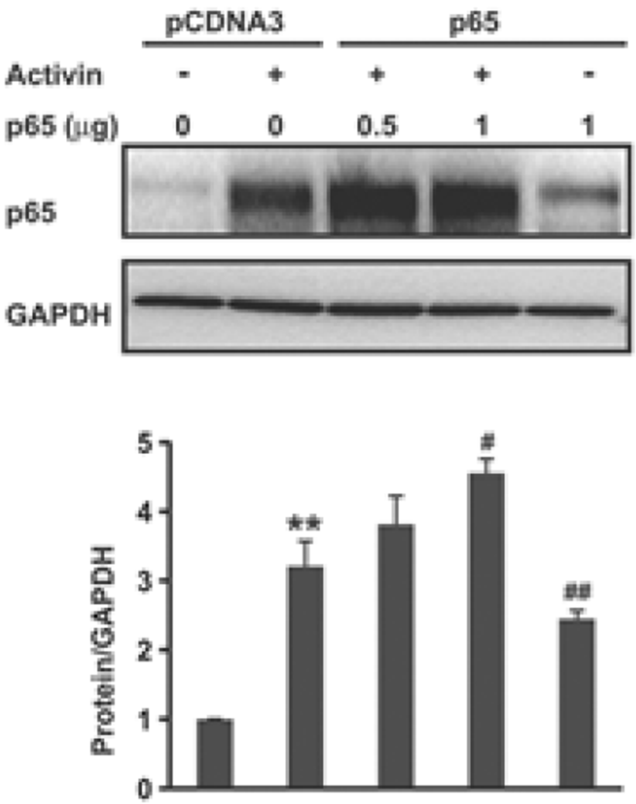

Supplementary Figure 5: Transfection efficiency of p65 is determined by western blotting. FET colon cancer cells were electroporated with pCDNA3-p65 or the empty vector. 48h after transfection, the cells were stimulated for 24h by treatment with activin (25ng/ml). Overexpression of p65 was confirmed by western blotting from the cell lysates. Bands were quantified and presented as relative expression compared to GAPDH and error bars represent the standard deviation of three independent experiments. * p indicates versus pCDNA3; # p indicates versus activin. # $p < 0.05$, **/## $p < 0.01$.

Supplementary Table 1: Primer list (H.sapiens)

RT-PCR Primers		
**Gene**	**Forward**	**Reverse**
**MDM2**	CAGCAGGAATCATCGGACTCA	ATCACTCTCCCCTGCCTGAT
**L19**	CCGAGCGAGCTCTTTCCTTT	CCGCTTACCTATGCCCATGT
**ChIP RT-PCR primers**		
**NFkB sites**	**Forward**	**Reverse**
**NFkB1** (-1084-918)	CAGGATTGTCCTGCCTCAGCCTCC	GGGTAGGCCGAGGCGGGTGGATCA
**NFkB2** (-1669-1470)	TCATCCAGGGTCAAGCACTGAGTC	CCTTCCCTTCTCCCGCTTCCCAGC
**NFkB3** (-3026-2710)	CCTGATTCTGATTTTTCAGACATCAC	GTATGACTCAGTGTCCACCTAAAGCA
**NFkB4** (-3190-3008)	GGGCCTCTTGAGGATGCGGACCTGTG	GAGGAGGTTGCTGAGAGCTGGGAGGAG
**NFkB5** (-3310-3160)	GGCGCTGCCCAGGATCCGCTTCCTGG	TCCCAAAGTGCCCATGATTCAGG
